# Comment on “Endothelial Protein C Receptor (EPCR), Protease Activated Receptor-1 (PAR-1) and Their Interplay in Cancer Growth and Metastatic Dissemination” *Cancers* 2019, *11*, 51

**DOI:** 10.3390/cancers11030374

**Published:** 2019-03-16

**Authors:** Giulia Pontarollo, Florentina Melzow, Christoph Reinhardt

**Affiliations:** 1 Center for Thrombosis and Hemostasis (CTH), University Medical Center Mainz, Johannes Gutenberg University of Mainz, Langenbeckstrasse 1, 55131 Mainz, Germany; Giulia.Pontarollo@unimedizin-mainz.de (G.P.); florentina@melzow.de (F.M.); 2 German Center for Cardiovascular Research (DZHK), Partner Site RheinMain, 55131 Mainz, Germany

Dear Editor,

Although the interplay between tumor progression and blood coagulation has been recognized since the milestone works by Bouillaud and Trousseau [1,2], the molecular mechanisms remain elusive. The recent review article by Marek Z. Wojtukiewicz and co-workers [3] provides a comprehensive overview of the impact of endothelial protein C receptor (EPCR; CD201) and protease-activated receptor-1 (PAR-1), traditionally related to the blood coagulation cascade, on cancer growth and metastasis. The authors propose that EPCR and PAR-1 share a common activated Protein C (APC)-dependent pathway in promoting tumor growth and dissemination. The APC/EPCR/PAR-1 axis is well-described as a major pathway promoting cancer progression, and the therapeutic potential of regulating this signaling cascade is proposed in this review article. The recombinant form of human APC turned out to be beneficial in severe sepsis cases [4,5,6], although the reduction of mortality was questioned [7]. However, the possibility of transferring APC cytoprotective effects to cancer models encounters some obstacles, due to its short half-life and the risk of bleeding complications [8].

In the first part of the paper, EPCR and PAR-1 canonical signaling pathways are detailed (Figure 1). Endothelial protein C receptor is mainly present on the vascular endothelium [8,9], and after binding of its physiological ligand protein C (PC), the PC–EPCR complex strongly accelerates PC conversion to active APC by the thrombin–thrombomodulin (TM) complex. The anticoagulant APC, in turn, (i) degrades and inactivates FVa [10] and FVIIIa [11], thus dampening the thrombin-dependent amplification of the blood-clotting cascade, and (ii) promotes fibrinolysis by neutralizing plasminogen activator inhibitor-1 (PAI-1) [12]. In addition, the observation that EPCR interacts with the γ-carboxyglutamic acid-rich (Gla) domain of PC/APC through recognized regions, prompted researchers to look for additional binding partners. Actually, EPCR interacts with FVII/FVIIa with an affinity similar to PC/APC, while its binding to FXa remains controversial [13,14,15]. The biological significance of the FVIIa–EPCR interplay remains somewhat obscure, although its primary effect is the scavenging to APC anticoagulant effects, thus increasing basal levels of blood coagulation. In analogy to PC/APC, binding of FVIIa to EPCR promotes the endocytosis of the ligand–receptor complex via a dynamin- and caveolar-dependent mechanism. After internalization into a recycling compartment, EPCR is targeted back to the apical side, while FVIIa is able to translocate to the basal surface, being cleared from the bloodstream [16]. Of note, also other components of the protein C pathway are internalized. For instance, PC inhibitor (PCI) undergoes membrane translocation through a phosphatidylethanolamine-dependent mechanism. In macrophages, phosphatidylethanolamine allocation within the PCI structure allows the translocation of the binary lipid–protein complex into the cytoplasm, where PCI may enhance phagocytosis of bacteria [17]. In summary, the cellular trafficking of the PC pathway components and the interchange binding of other coagulation proteases is an interesting aspect that deserves closer attention.

More interestingly, the authors give insights into other “hidden” functions of EPCR that place this receptor at the interface between hemostasis and inflammation. Besides endothelial cells, EPCR is expressed on a variety of cell types, including dendritic cells [5], leukocytes, epithelial cells [18], and hematopoietic stem cells [19], suggesting a role for this receptor in immune regulation. The common evolutionary origin of blood clotting factors and immune receptors is well established [20]. Surprisingly, EPCR shares a structural resemblance with the CD1/major histocompatibility complex superfamily, both in its primary sequence [21] and in its three-dimensional conformation [22]. In mast cells, EPCR was recently implicated as a non-conventional antigen-presenting molecule, involved in γδ T cell activation [23]. However, the exact role of the antigen-presenting function of EPCR remains enigmatic.

Along with EPCR, in recent years, novel functions have been emerging for PC/APC, placing it center stage in cellular signaling. Once in its active form, APC may exploit the canonical anti-coagulant and fibrinolytic functions or—in complex with EPCR—it may attack and activate the PAR-1 receptor. The unexpected role of the APC–EPCR complex in PAR-1 proteolysis was uncovered by Wolfram Ruf and co-workers [24], and since then, several studies unraveled the cytoprotective, anti-inflammatory, and anti-apoptotic functions of this pathway [25]. Protease-activated receptors (PARs) are G-protein coupled receptors broadly expressed on a variety of cell types, mediating pleiotropic effects through the interaction with different ligands. In particular, PAR-1 (i.e., one of the four PAR isoforms described) [26] is present on nearly all vessel wall and blood cell types, with the exception of erythrocytes [27], playing a central role in vascular development [28]. The major PAR-1 agonist is thrombin, which, by attacking the N-terminal extracellular domain at the peptide bond Arg41–Ser42, generates a novel N-terminus (tethered ligand) that interacts with the receptor body, mediating intracellular (G12/13, Gq) signaling. Thrombin-induced PAR-1 activation results in the production of inflammatory cytokines, chemokines, growth factors, and bioactive lipids. These molecular determinants, in addition to the direct effects promoted by thrombin, contribute to cellular adhesion and endothelial barrier disruption, with an overall pro-inflammatory outcome [29,30]. In this context, plasmin may desensitize thrombin-dependent Ca^2+^ signaling by removing the N-terminal tethered ligand from the PAR-1 receptor [31]. Besides this traditional cascade, PAR-1 is subjected to proteolysis at Arg46 by APC in the APC–EPCR complex, thus stimulating Gi signaling pathways, associated with anti-inflammatory and cytoprotective effects (biased agonism concept) [32]. Interestingly, the APC–EPCR complex was demonstrated to improve epithelial barrier function in the colon, protecting against the onset of dextran sulfate sodium-induced colitis in mice [18]. This opposite, yet complementary function of PAR-1 is partially explained by the contribution of caveolin-1 binding to EPCR, modulating G-protein signaling, and can be alternatively accomplished through the β-arrestin pathway [33]. Various aspects of the enormous complexity of PAR signaling (Figure 1) is conceptionally grasped in the review article by Wojtukiewicz et al. [3].

While the expression profile of PARs in different tissues is well defined, the tissue-specific signaling function of these receptors is poorly resolved. In particular, while there is a wealth of knowledge on PAR-1 signaling in endothelial cells and its regulatory role in vascular permeability and tumor intravasation [34], it is interesting to clarify the role of epithelial PARs, of primary interest in carcinogenesis and tumor progression. Although proteases are generally known to be involved in basement membrane remodeling of the tumor microenvironment [35], little is known about protease receptors. Several investigations tried to address the epithelial tissue factor (TF), PAR-1 and PAR-2 as carcinogenesis biomarkers. Recent studies demonstrated that TF and PAR-2 are overexpressed in colorectal carcinoma [36], while TF-mediated PAR-2 activation is an established pro-migratory, pro-invasive, and angiogenetic factor in breast cancer [37]. On the other hand, the impact of PAR-1 in tumor invasion and metastasis (e.g., in colorectal carcinoma, breast, and ovary cancer) is still a matter of debate [36,37,38]. Actually, PAR-1 and EPCR have been detected on multiple carcinoma subtypes, and paradoxically, both the proinflammatory and the cytoprotective effects, mediated by the APC/EPCR/PAR-1 axis, may be crucial for malignant cell proliferation and dissemination. To support this hypothesis, Wojtukiewicz et al. [3] present various studies, performed on in vivo and in vitro models. For instance, TF-dependent activation of PAR-1 led to increased expression of angiopoietin-1 (Ang-1) and activation of its receptor tyrosine kinase (Tie-2). The latter, in turn, is overexpressed on the endothelium of vascular “hot spots” in human breast cancer specimens [39], while the Ang-1/Tie-2 pathway promotes the remodeling of capillary endothelial cells in small intestinal villus structures [40]. Conversely, according to recent research, endogenous APC could limit cancer cell extravasation through endothelial barrier enhancement in an EPCR and PAR-1-dependent process that involves phosphorylation of the sphingosine-1-phosphate receptor-1 [41]. A tentative explanation for this open issue is given in the second part of the manuscript [3], in which the authors list the factors that may be involved in the APC/EPCR/PAR-1 regulation. The determinants affecting PAR-1 signaling include: (i) modulation of PAR-1 response by G-proteins (i.e., G12/13, Gq, Gi) or β-arrestin pathways; (ii) stimulation of EPCR and PAR-1 by TF, FVIIa, and FXa; (iii) effect of TM; and (iv) impact of the microbiota and its secreted proteases. Finally, the effects of APC/EPCR/PAR-1 on hematopoietic stem cells and neuronal development are described [3].

A fascinating aspect that needs further investigation is the effect of the commensal gut microbiome on tumor growth and dissemination. The huge diversity of the 10^14^ microorganisms colonizing the gastrointestinal tract is a non-negligible environmental factor, modulating multiple signaling pathways. Besides its trophic and metabolic functions, the gut microbiota has an impact on cancer initiation, progression, and even on anticancer therapies [42,43,44]. Recent studies indicate that approximately 18% of human cancer cases are directly caused by chronicity of bacterial infections (e.g., *Helicobacter pylori* infection is a significant risk factor of stomach cancer) [45,46]. In this context, the gut microbiota plays a protective role via the occupation of ecological niches and training of the host immune system [47]. Vice versa, an alteration of the well-balanced microbial ecology (in abundance and/or composition) may be detrimental for the host. Dysbiosis, caused by environmental factors like diet, antibiotics, infectious disorders, immune system alteration or even anticancer therapies may indeed contribute to cancer progression [48].

Gut resident bacteria could potentially affect the APC/EPCR/PAR-1 axis by modulating PARs (i) expression and (ii) activation. In the small intestine, the gut microbiota is known to upregulate PAR-1 expression and to trigger tissue factor-dependent coagulation factor signaling [40]. Proteases secreted from resident microbes may activate PARs via direct proteolytic action, as shown for PAR-2 through *Enterococcus faecalis* gelatinase [49], or indirectly through non-canonical activation of human prothrombin [50,51].

In conclusion, the review article by Wojtukiewicz and co-workers [3] describes the molecular interactions between EPCR and PAR-1 along with the development of cancer and metastasis, exploiting well-established concepts, but also giving insights on novel findings (Figure 1). The unraveling of yet unexplored biochemical pathways may detect novel therapeutic targets, paving the way to improved anticancer therapies.

## Figures and Tables

**Figure 1:**
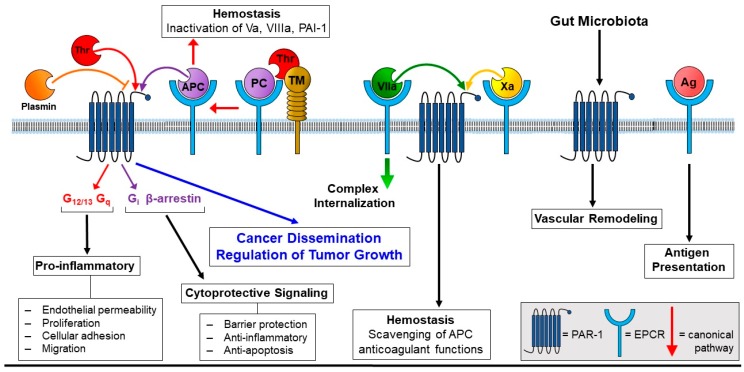
Moonlighting functions of the endothelial protein C receptor (EPCR). In addition to its canonical role as a cofactor in the anticoagulant protein C pathway, localized to the vascular endothelium, EPCR is also expressed on other cell types and ascribed new roles in antigen presentation, epithelial permeability regulation, cancer cell evasion, anti-apoptotic effects, and anti-inflammatory protease-activated receptor-1 (PAR-1) signaling. Abbreviations: Thr, thrombin; PC, protein C; APC, activated protein C; Va, Vllla, Vlla, Xa, active coagulation factors; Ag, antigen; TM, thrombomodulin; PAI-1, plasminogen activator inhibitor-1.
